# Influence of Twin Screw Extrusion Conditions on MWCNT Length and Dispersion and Resulting Electrical and Mechanical Properties of Polycarbonate Composites

**DOI:** 10.3390/polym16192694

**Published:** 2024-09-24

**Authors:** Petra Pötschke, Tobias Villmow, Beate Krause, Bernd Kretzschmar

**Affiliations:** Leibniz-Institut für Polymerforschung Dresden e.V. (IPF), Hohe Straße 6, 01069 Dresden, Germanykrause-beate@ipfdd.de (B.K.); bkretzsch@online.de (B.K.)

**Keywords:** polycarbonate, multiwalled carbon nanotubes, melt mixing, dispersion, electrical conductivity, nanotube length, mechanical properties

## Abstract

The processing conditions were varied during the production of polycarbonate-based composites with the multiwalled carbon nanotubes (MWCNTs) Baytubes^®^ C150 P (Bayer MaterialScience AG, Leverkusen, Germany), by melt mixing with an extruder on a laboratory scale. These included the screw design, rotation speed, throughput, feeding position and MWCNT content. Particular attention was paid to the shortening of the MWCNT length as a function of the conditions mentioned. It was found that there is a correlation between the applied specific mechanical energy (SME) during the melt mixing process and MWCNT dispersion, which was quantified by the agglomerate area ratio of the non-dispersed nanotubes based on optical microscopic analysis. The higher the SME value, the lower this ratio, which indicates better dispersion. Above an SME value of about 0.4 kWh/kg, no further improvement in dispersion was achieved. The MWCNT length, as measured by the quantitative analysis of TEM images of the MWCNTs dissolved from the composites, decreased with the SME value down to values of 44% of the original MWCNT length. At a constant loading of 3 wt.%, the tensile strength and tensile modulus were almost independent of the SME, while the elongation at break and notched impact strength showed an increasing trend. The variation in the feeding position showed that feeding the MWCNTs into a side feeder led to slightly better electrical and mechanical properties for both types of MWCNTs studied (Baytubes^®^ C150 P and Nanocyl™ NC7000 (Nanocyl S.A., Sambreville, Belgium)). However, feeding into the hopper led to better CNT dispersion with Baytubes^®^ C150 P, while this was the case with Nanocyl™ NC7000 when feeding into the side feeder. The screw profile had an influence on the dispersion, the MWCNT length and the electrical resistance, but only to a small extent. Distributive screws led to a greater shortening of the MWCNT length than dispersive screws. By varying the MWCNT content, it was shown that a greater MWCNT shortening occurred at higher loadings. Two-stage masterbatch dilution leads to stronger shortening than composite production with direct MWCNT incorporation.

## 1. Introduction

In polymer nanocomposites, the full potential of the nanofillers can be only exploited if the particles are dispersed on a nanoscale and suitably distributed in the matrix. Then, a high interfacial area is created which at the same time means that the polymer chains around the nanoparticles are restricted by the contact with the filler surface and display different properties than in the matrix. When the property of electrical conductivity of carbon nanoparticles should be introduced into the composite, then a network of conductive fillers has to be established. This can be formed either by direct contact between neighboring nanoparticles or using hopping and tunneling effects between neighboring filler particles.

Carbon nanotubes (CNTs) provide a special challenge in dispersion into individualized nanoparticles. Due to their synthesis process and their cylindrical shape, they always exist as primary agglomerates, which can have quite different morphologies [1]. Their dispersibility mainly depends on their structural characteristics, like their length, diameter, waviness, branching, defect density and surface characteristics including functional groups. Their achievable state of dispersion in different media depends in addition on the matrix viscosity and surface energy properties, resulting in different interactions, but also very strongly on the processing conditions. Depending on all these influencing factors, not only can the state of dispersion be influenced significantly, but also the length of the CNTs can be reduced in different amounts.

Many investigations were performed on the influence of melt mixing conditions on dispersion and electrical properties on a small scale, such as using microcompounders. For polycarbonate (PC)-based composites with multiwalled CNTs (MWCNTs), Alig et al. [2] performed dielectric measurements on composites prepared with the masterbatch approach using different mixing times and rotation speeds. They concluded that near the percolation threshold (here, 1.0 wt.%), an increase in mixing time could improve dispersion in such a way that non-percolated systems got percolated. Decreased conductivity values when increasing the mixing speed or mixing time at a higher loading (1.5 wt.%) were explained with more pronounced nanotube shortening. However, no measurements on the nanotube length were performed. Lin et al. [3] compared three different types of miniature mixers when diluting an MWCNT masterbatch and measured the MWCNT length after processing by applying atomic force microscopy (AFM). Therefore, the composites were dissolved in methylene chloride, filtered, treated with sodium–hydroxyl–ethanol solution under sonification, washed, dried and dispersed in chloroform. Silicon wafers were dip coated in these dispersions and studied. Using this procedure, it was shown that after the melt mixing, the MWCNTs possessed length values between 66% and 74% of their initial lengths. This indicated that shortening occurred, however it was not very different when using the different small-scale mixers. The electrical percolation thresholds were lower for the composites produced with custom-built mixers (APAM and MBM) than with the commercial DACA microcompounder, even if the dispersion (investigated by TEM) was best for the DACA mixer. Thus, it was concluded that small agglomerates may help to form the electrical network. 

In polyamide (PA)-based composites prepared by direct incorporation of MWCNT Nanocyl™ NC7000 nanotubes, Krause et al. [4] varied the mixing speed and mixing temperature and studied electrical percolation and dispersion. At constant mixing conditions, PA66 as a matrix resulted in a lower electrical percolation threshold than PA6, and increasing the screw speed in PA6 increased the threshold. The dispersion of 5 wt.% MWCNTs in PA6 improved when increasing the mixing speed. After calculating the mixing energy input for each mixing process, the increase in the state of dispersion with mixing energy was accompanied by an initial decrease in electrical volume resistivity followed by an increase when performing the mixing with a higher-energy input. Also, it was concluded that more pronounced CNT shortening at higher-mixing energy input may have been the reason for that resistivity increase. 

In a study on high-impact polystyrene by McClory et al. [5], with MWCNTs from a Chinese producer, variation in screw speed (20 rpm to 150 rpm) at otherwise constant conditions resulted in the best dispersion at an intermediate screw speed (100 rpm). At higher speeds, the percolation concentration was significantly increased, which was assigned to a lower disentanglement of MWCNT agglomerates at such high speeds.

Kasaliwal et al. [6,7] studied polycarbonates with MWCNT Baytubes^®^ C150 HP in detail to determine the dispersion mechanisms of primary nanotube agglomerates by varying the mixing speed, mixing time and melt temperature during small-scale compounding. The area ratio of the remaining agglomerates, as determined from thin sections using transmission light microscopy, was used was used to quantify the state of dispersion. From the relative change in the area ratio vs. mixing time at different mixing speeds and a proposed model, the time coefficients of the two mainly considered processes of rupture and erosion were calculated, from which the fractions of rupture and erosion mechanisms during agglomerate dispersion were estimated. At low mixing speeds, dispersion was found to be governed by both mechanisms, whereas the dominance of the rupture process increased with increasing mixing speed. Further, the relationship between electrical resistivity and dispersion was studied, indicating that a dependency on the amount of dispersed nanotubes was found only in a certain range of states of dispersion. The electrical resistivity of the composites decreased up to a specific mechanical energy (SME) of 1400 J/cm^3^, whereas at higher mixing energies (up to about 55,000 J/cm^3^), reached by either enhancing the mixing speed or mixing time, a plateau with low electrical resistivities was found.

In another study by Kasaliwal et al. [8,9], the molecular weight of the PC and thus the melt viscosity of the matrix material was changed, resulting in better dispersion at higher matrix viscosity when using constant mixing conditions but worse considering comparable shear stress values. To selectively study the influence of the molecular weight, the melt mixing temperatures of the three used PCs were adjusted to have similar viscosities, and melt mixing was performed at a constant mixing speed. As investigated on two viscosity levels, the composites based on the low-molecular weight matrix showed smaller undispersed primary agglomerates as compared to composites with higher molecular weight matrices. This result was discussed to indicate the role of matrix infiltration in primary nanotube agglomerates as the first step of dispersion, which is faster at lower molecular weights or lower matrix viscosities. The resistivity values of composites prepared using low-viscosity matrices were lower than those of composites from a high-viscosity matrix.

Such an influence of the matrix viscosity was also studied for other polymer matrices. Socher et al. presented a study comparing the matrix polymers polyamide 12, polybutylene terephthalate, PC, poly(ether ether ketone) and low-density polyethylene with three viscosity levels each in composites with MWCNT Baytubes^®^ C150 P [10]. Huge differences in the electrical percolation thresholds were found using the same polymer matrix at different viscosity grades. The composites with the low-viscosity matrix always resulted in the lowest percolation threshold. The state of primary MWCNT agglomerate dispersion increased with matrix viscosity due to the higher input of mixing energy. This study also focused on the effect of the shortening of nanotubes due to breaking during processing, using two viscosity grades of PC. To evaluate such influences, a more sophisticated method was developed by Krause et al. [11] using the TEM analysis of single nanotubes dissolved from the composite sample that was applied in this study. The effect of nanotube shortening was quantified using two different viscosity grades of PC. Due to the higher mixing energy input, the nanotube shortening was more pronounced in the high-viscosity matrix, which partially explained the higher percolation threshold. 

The relationships between the rotation speed during small-scale mixing (varied between 25 rpm and 400 rpm) and different properties of composites of polycaprolactone (PCL) and 0.5 wt.% Nanocyl™ NC7000 was studied in a comprehensive manner by Pötschke et al. [12]. The properties studied included the molecular weight of PCL after processing, PCL melting and crystallization behavior, MWCNT macrodispersion, MWCNT length distribution, electrical resistivity, and oscillatory melt rheological properties of the composites. The MWCNT dispersion was improved with increasing rotation speed but leveled off starting at about 100 rpm. The molecular weight of the PCL as well as crystallization and melting behavior did not change significantly when varying the rotation speed. The storage modulus G’ and complex viscosity η* at 0.1 rad/s increased up to a rotation speed of about 75 rpm, illustrating improved dispersion. When the speed values were further increased, both values decreased. This was attributed to more pronounced MWCNT shortening, as quantified by TEM. Both effects, improved dispersion and nanotube shortening, were reflected in the electrical resistivity of compression-molded samples, which showed a minimum of resistivity at the rotation speed of 75 rpm, corresponding to a specific mechanical energy input of 0.47 kWh/kg. Mayoral et al. studied the influence of mixing speed (50 to 200 rpm), mixing time (1–30 min) and MWCNT loading (0.5 to 2.0 wt.%) in the batch mixing of polypropylene (PP)–MWCNT composites, with a focus on the agglomerate sizes and their distributions, melt rheology and electrical resistivity. Concerning MWCNT dispersion, the highest rotor speed at the longest mixing time resulted in being the best, whereas the highest electrical conductivity was achieved at a medium speed and mixing time. The effects were explained with a suitable hierarchical arrangement of MWCNT agglomerates, smaller MWCNT bundles and individual MWCNTs, leading to the best conductive network; CNT shortening was not considered [13]. More examples of the influence of material properties and small-scale mixing conditions on dispersion and electrical and mechanical properties can be found in the literature [14], whereby the study of CNT shortening during processing is quite limited. 

Even if the main results of such small-scale mixing experiments can be applied to laboratory twin screw extrusion, as commonly used in composite fabrication by melt mixing processes, much less studies have concerned the influence of twin screw processing conditions on the CNT dispersion, nanotube length and electrical resistivity or mechanical properties of the composites. The first examples were given by Villmow et al. [15] on poly(lactic acid) (PLA) with Nanocyl™ NC7000, who studied the influence of the screw profile, temperature profile and rotation speed (100 rpm and 500 rpm) using a Berstorff ZE25 extruder (KraussMaffei Berstorff, Laatzen, Germany) on the state of dispersion in masterbatches containing 7.5 wt.% and 15 wt.% MWCNT. These masterbatches were then diluted to composites with 0.75 wt.% MWCNT under varied process conditions. Interestingly, the state of MWCNT dispersion in the diluted composites was predominated by the state of filler dispersion in the masterbatches, independent of the mixing conditions during dilution. It was found that a high rotation speed that still ensured a certain residence time of the melt combined with a screw profile containing mainly mixing elements were convenient to disperse and distribute the MWCNTs in the PLA matrix as well during the masterbatch production as the dilution step. The temperature profile showed less influence; however, a profile with increasing temperatures along the screw resulted in slightly better nanotube dispersion. In their study, it was shown that differential scanning calorimetry could be used to judge the state of MWCNT dispersion in the masterbatches where microscopic methods had problems in distinguishing different states of dispersion. As more CNTs are dispersed, the higher the nucleating action of the CNT surfaces on the PLA. 

In a later study by Villmow et al. [16], similar processing was adapted to PCL melt mixed with Nanocyl™ NC7000, with a special focus on the influence of screw configuration on the residence time, SME and the resulting macroscopic CNT dispersion in masterbatches containing 7.5 wt.%. In addition, the rotation speed and throughput were varied and samples along the screw were taken. The main output of their study was that the processing conditions had a strong influence on the residence time (t_R_) of the extrudates and on the CNT dispersion. Both an increase in the rotation speed and throughput resulted in a decrease in t_R_, whereas the use of back-conveying elements and the extension of the processing length showed the opposite effect. With increasing rotation speed, higher SME inputs and a significant increase in CNT dispersion were observed, whereas an increase in throughput resulted in worse dispersion. The use of distributive screw configurations containing mixing elements can further promote CNT dispersion. The best dispersion was found when using an extended distributive screw having a length-to-diameter ratio (L/D) of 48 instead of 36.

Finally, using the same equipment, the influence of the feeding position of the MWCNTs on the dispersion and properties of composites was also studied. Müller et al. [17] investigated polypropylene-based composites with both, Baytubes^®^ C150 P and Nanocyl™ NC7000. Both MWCNT materials were fed at selected concentrations either in the hopper or using a side feeder under otherwise identical extrusion conditions. The results led to the conclusion that the more compact Baytubes^®^ C150 P agglomerates should be added into the hopper, as the dispersion was better, the electrical resistivities measured on compression and injection-molded samples were lower, and the elastic modulus, yield strength and impact strength were higher as compared to side feeding. On the other hand, for the more loosely packed Nanocyl™ NC7000 agglomerates, addition using the side feeder led to better dispersion, lower electrical resistivity and higher mechanical properties. This agrees with the different dispersibility and agglomerate strengths of both MWCNT materials as shown in a dispersion study by Krause et al. [18] and illustrates that the structure of the primary agglomerates of MWCNTs has to be taken into account when optimizing processing conditions.

A study by Benedito et al. [19,20] used commercial or self-prepared thermoplastic polyurethane (TPU)–MWCNT masterbatches (15 wt.% NC7000) to dilute them in the co-rotating twin screw extruder Coperion ZSK25 under different compounding parameters. As shown in [19], using light microscopy images on 1 wt.% composites, the dispersion was significantly better in composites prepared at 600 rpm than in those made at 200 rpm. Dynamic rheology and dynamic mechanical analysis (DMA) were used to quantify differences at varied loadings. In [20], a masterbatch was prepared with a screw, comprising a combination of high-shear dispersive elements, distributive elements and mixing elements under a high screw speed of 600 rpm. The dilution was performed using the named screw and one with mainly transport elements, resulting in a lower residence time and mechanical energy at speeds of 200 rpm and 600 rpm. The screw design was shown to have a significant impact on the MWCNT dispersion, with significantly better dispersion when using the screw design with a higher energy input. At higher screw speeds, better dispersions were achieved, as quantified using a term called “agglomeration density”. On the other hand, the samples with the worser dispersion, prepared at a low speed or using the low-mechanical energy screw design showed higher electrical conductivity. The shortening of MWCNTs due to breakup was hypothesized. 

Another example is a later study by Mack et al. [21] on PC-based composites with 1, 3 and 5 wt.% MWCNT Nanocyl™ NC7000 processed in a twin screw extruder Leistritz ZSE 27HP-52D with two different screw configurations, screw speeds and throughputs, resulting in different SMEs at different conditions. Therefore, screw 1 was designed to have mainly kneading elements and transportation elements and screw 2 had some kneading elements replaced by other elements. The volume resistivity of the injection-molded PC-1 wt.% MWCNT composites varied only slightly with the conditions, whereas at 3 wt.% loading, the resistivity was independent of the process conditions. The modulus and strength of the composites were enhanced with filler addition, whereby at 5 wt.% loading, the strengths of the composites produced at higher SMEs (both screws, screw speed of 1100 rpm and throughput of 7.5 kg/h) were higher than those produced at lower SMEs (both screws, lower rotation speed of 500 rpm and higher throughput of 12.5 kg/h). The highest value was achieved for screw 2 at the lower throughput and the higher screw speed, which however, was not the sample that had experienced the highest SME. At the same filler loading, composites processed with higher SMEs showed, at all MWCNT concentrations, higher notched impact strength values which were assigned to the better filler dispersion. The same group also studied composites based on polystyrene (PS) and Baytubes^®^ C150 P [22]. Here, 1, 2, 3, 5 and 7.5 wt.% MWCNTs were incorporated into PS in the twin screw extruder at varied speeds, throughputs and extruder barrel temperatures at two levels each. The higher SME at an enhanced processing speed was found to have the largest effect on enhancing nanotube dispersion. The electrical volume resistivity was significantly lower in the 2 wt.% MWCNT samples processed at 1100 rpm compared to those produced at 500 rpm. The influence of screw speed on the properties using the same equipment was also studied for composites based on polypropylene and 2 wt.% MWCNT Nanocyl™ NC7000 [23]. Better dispersion was found at the higher speed combined with a higher modulus, slightly higher tensile strength and slightly reduced notched impact strength in injection-molded composites. In 2023, Ezat et al. studied the effect of screw configuration using the intermeshing twin screw extruder PRISM-TSE-16-TC, using PP and acid-purified MWCNTs from Cheap Tubes, modified with a maleic anhydride-grafted PP as a compatibilizer [24]. Among the three designed screws, the high-intensity configuration with a higher number of kneading blocks achieved the best dispersion and lowest percolation concentration at 2 wt.%, compared to a standard screw and the screw designed to establish chaotic mixing conditions. The tensile properties were only slightly affected by the screw configuration. The tensile modulus was slightly higher when using the medium-intensity (standard) screw. Also, the tensile strength of the PP was most improved by the MWCNTs when incorporated using that standard screw.

In all these laboratory-scale studies, a low focus was put on the effect of the extrusion step on the CNT length, even if a possible shortening was partially named as the reason for unexpected results (e.g., [20]). However, to understand the relationships between the processing conditions, morphology and properties of nanocomposites completely, the change in the initial length of the reinforcing nanotubes and the effect of processing conditions on that phenomenon should also be considered. Studies are needed not only on small-scale mixing devices, but also on laboratory-scale extrusion (LSE). In addition to the already named studies made for small-scale mixing experiments, Krause et al. [25] studied the shortening of MWCNTs Nanocyl™ NC7000 ball-milled at different times (5 h, 10 h) after their melt extrusion with PC on a laboratory scale. They found that after melt compounding, the mean CNT lengths were shortened to 31%, 50% and 66% of the initial lengths of NC7000, NC7000-5 h and NC7000-10 h, respectively. In small-scale experiments, Liebscher et al. [26] compared the lengths of NC7000 MWCNTs in PC composites with 0.5 and 1.0 wt.% CNTs when using two different mixing conditions. It was found that the shortening was slightly higher when harsher mixing conditions were applied, such as a higher rotation speed and longer mixing time. For composites with 0.5 wt.% MWCNTs, after the harsher conditions, the initial CNT length before compounding was reduced to 70%, whereas after the milder conditions, it was reduced to 76%, based on the x_50_ values. From comparing samples with 0.5 wt.% and 1.0 wt.%, the CNT length shortening was more pronounced at the higher loading. In addition, the authors explored that even if the CNTs preferred the PC component, blending with a second polymer, namely styrene–acrylonitrile copolymer (SAN), to obtain co-continuous blend morphologies further reduced the CNT length [26]. Finally, in the blends, the lengths were only 27% and 30% of the initial length at harsher and milder conditions for blends containing 0.5 wt.% CNTs, respectively. In contrast, Guo et al. found, for small-scale mixed PS/poly (methyl methacrylate) (PMMA) blends with MWCNTs showing matrix droplet morphologies, that relatively long MWCNTs broke less in the polymer blends than in the neat polymer droplet material [27]. This resulted in a reduction in the percolation threshold. This breakage depended on the amount of the component which the CNTs preferred (here, PMMA) and was lower when filled PMMA formed the droplets [27].

Based on the lack of information about CNT length changes in laboratory-scale melt-compounded composites, this study is intended to show comprehensive results obtained on PC-based composites with industrial multiwalled carbon nanotubes. The composites were obtained by laboratory-scale extrusion (LSE) compounding with varied rotation speeds, throughputs, feeding positions and screw profiles. Therefore, a special focus was laid on the study of the CNT length after compounding, which so far has not been systematically studied. In addition, the state of dispersion was studied using light microscopy, and the electrical and selected mechanical properties are presented.

## 2. Materials and Methods

### 2.1. Materials and Processing

As the polycarbonate (PC), medium-viscosity general-purpose Makrolon^®^ 2600-grade (Bayer MaterialScience AG, Leverkusen, Germany), intended for injection molding with a melt volume–flow rate of 12.0 cm^3^/10 min (1.2 kg, 300 °C [28]), was applied. As for the molecular weight of this material, a previous study indicated an M_n_ value of 9500 g/mol, an M_w_ value of 26,200 g/mol g/mol and a polydispersity index of 2.76 [9]. Further properties of this material are given in its datasheet [28].

As for the multiwalled carbon nanotubes (MWCNTs), the widely used, commercially available Baytubes^®^ C150 P (Bayer MaterialScience AG, Leverkusen, Germany), with a carbon purity of ~95%, bulk densities of 120–170 kg/m^3^, agglomerate sizes of 30–550 µm [18], a mean diameter of 10.5 nm and a mean length x_50_ of 770 nm [29], were used. From these data, a mean aspect ratio of 73 was calculated. More information about this material can be found in its datasheet [30]. In addition, the commercially available MWCNT material Nanocyl^TM^ NC7000 (Nanocyl S.A. Sambreville, Belgium), with a carbon purity of >90%, a bulk density of 66 kg/m^3^ [31], an agglomerate size of >675 µm [18], a mean diameter of 10.0 nm and a mean length x_50_ of 1341 nm [29], was also applied for comparison in part of the study. It had a mean aspect ratio of 134. For more information about the material, please refer to its datasheet [32]. The electrical conductivity of these MWCNT powdery materials was measured using a powder cell, and at a pressure of 30 MPa, values of 15 S/cm for the Baytubes^®^ C150 P and of 24 S/cm for the Nanocyl^TM^ NC7000 were obtained [33]. Scanning electron microscopic images of the initial agglomerates of Baytubes^®^ C150 P and Nanocyl^TM^ NC7000 are given in [34]. More information about the structural details of the Baytubes^®^ and Nanocyl^TM^ MWCNT products can be found in a detailed study by Tessonnier et al. in [35].

To study the influence of the processing conditions in laboratory-scale extrusion (LSE), in a first set polycarbonate composites filled with 3 wt.% Baytubes^®^ C150 P were prepared using a co-rotating twin screw extruder ZE25 (KraussMaffei Berstorff, Laatzen, Germany), with an L/D ratio of 48 and at a mean barrel temperature of 260 °C. A number of variations were carried out. On the one hand, using a fixed screw configuration, the influence of rotation speed, varying between 100 and 1000 rpm, and the effect of the throughput, varying for all rotation speeds between 5 and 15 kg/h, were investigated. PC pellets and the powdery MWCNT material were fed simultaneously into the hopper by gravimetric dosing. To study the influence of the feeding position, at a rotation speed of 500 rpm and a throughput of 5 kg/h, the Baytubes^®^ C150 P or Nanocyl^TM^ NC7000 were added either in the hopper together with PC granules or into the side feeder attached at an L/D of 14. In a second set, different screw configurations (SC, see Table 1) adapted from Ref. [16] were used, whereby the throughput was set to 5 kg/h and the rotation speed to 100 rpm. Pure PC pellets were also processed using SC-5 at 260 °C, 500 rpm and a 5 kg/h throughput. 

To relate the achieved physical properties to the energy that was introduced during the mixing process, the specific mechanical energy (SME) was calculated based on the torque values recorded during the compounding, using the following equation [18]:

(1) $$SME=\frac{2\pi N\tau }{\dot{m}}$$

where N is the mixing speed, τ is the mean torque value during compounding and ṁ is the throughput during the extrusion. 

The PC composites with 3 wt.% extruded in LSE by variation in throughput and rotation speed were further diluted to lower loadings between 0.5 und 2 wt.%, using the conical co-rotating twin screw microcompounder Xplore 15 (Xplore, MD, Geleen, The Netherlands), having an inner volume of 15 cm^3^. A mixing temperature of 280 °C, mixing speed of 50 rpm and mixing time of 5 min were applied. 

To study the influence of the MWCNT loading on the nanotube breakage of NC7000 in LSE, in a third set extruded composites based on PC Lexan 141R (Sabic, Riyadh, Saudi Arabia, melt volume–flow rate of 12 cm^3^/10 min at 1.2 kg and 300 °C, according to the datasheet [36]), with MWCNT Nanocyl^TM^ NC7000 (in short NC7000), were studied. Therefore, the Berstorff ZE25 extruder with an L/D ratio of 48 (SC-5) was again used with a throughput of 5 kg/h, rotation speed of 500 rpm and mean melt temperature of 260 °C. A masterbatch containing 7.5 wt.% CNTs was prepared, which was in a second extrusion step diluted to 4.0 and 2.0 wt.%. For comparison, composites filled with 2 and 4 wt.% NC7000 were prepared by direct CNT incorporation.

To measure electrical properties, the granules obtained from the extruded strands were compression-molded into plates (60 mm diameter, 0.5 mm thickness) using a Weber hot press (Model PW 40 EH, Paul Otto Weber GmbH, Remshalden, Germany). Compression molding was performed with a pressing temperature of 280 °C. The pressing speed was 6 mm/min, the pressing time 1 min and the pressing force was increased in steps up to 100 kN.

For mechanical testing, injection molding was performed using a Demag Ergotech 100/420–310 machine (Demag Plastics Machinery GmbH, Roßleben-Wiehe, Germany) at 290 °C melt temperature, injection speed of 35 mm/s and rotation speed of 200 rpm. Standard test bars for tensile testing and impact testing were produced.

### 2.2. Characterization Techniques

In order to access the state of nanotube dispersion, light microscopy (LM) in transmission mode was performed to investigate the macrodispersion of CNT agglomerates within the PC matrix. Thin sections with a thickness of 5 µm were cut from extruded strands using a microtome Leica 2055 (Leica Mikrosysteme Vertrieb GmbH, Bensheim, Germany) and were fixed on glass slides. The LM images were recorded using a BH2 microscope combined with a DP71 camera (both Olympus, Deutschland GmbH, Hamburg, Germany). The agglomerate area ratio A_A_ was determined from the light micrographs by calculating the ratio of the area of remaining agglomerates A_Agg_ to the total area of the micrograph A_0_ (~0.6 mm^2^) using the software ImageJ Version 1.43 g. In addition, the numbers of agglomerates were detected. Only agglomerates larger than 5 µm were considered for this calculation. For the quantification, at least six cuts from different positions were investigated for each sample, and the standard deviation between these cuts is given. 

**Figure 1:**
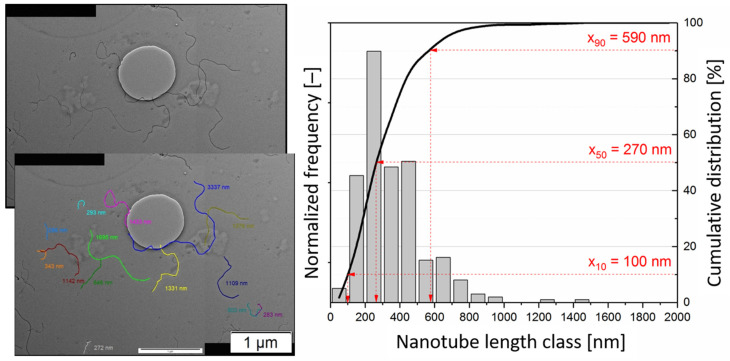
Schema of CNT length measurement: TEM micrograph containing the measured CNTs, a nanotube length histogram, the cumulative frequency distribution and the characteristic length values (here, with the example of processed Nanocyl^TM^ NC7000 MWCNTs).

For the determination of the nanotube length distribution after melt compounding in the polycarbonate matrix, a method described by Krause et al. [11] was used (see Figure 1). The polycarbonate composites containing 0.5 wt.% to 7.5 wt.% CNTs were dissolved in chloroform overnight followed by a treatment of 3 min in an ultrasonic bath. For the TEM investigations, a drop taken out from the freshly prepared dispersion (0.03 g CNT/l chloroform) was placed on a TEM grid with a carbon coating and dried in the air. In the TEM images collected with a Libra200 (Carl Zeiss Industrielle Messtechnik GmbH, Oberkochen, Germany), the nanotube lengths were measured on approximately 200–300 particles by applying the software SCANDIUM 5.1 (Olympus Soft Imaging Solutions GmbH, Hamburg, Germany). The full visible length of each separated nanotube, not touching the edge of the image, was thereby recorded by applying the polyline function. In order to measure the lengths of very long nanotubes, images were stitched together as needed. The results are given as number distributions with class sizes of 100 nm. To quantify the length distributions of the nanotubes, the typical distribution parameters (quantile) x_10_, x_50_ and x_90_ were calculated based on the cumulative frequency distributions, indicating that 10%, 50% and 90% of the nanotube lengths were smaller than the given value. For interpretation, mainly x_50_ was used. The TEM images on which the CNT lengths were measured are available as a dataset in [37].

To perform electrical volume resistivity measurements, rectangular samples (ca. 30 × 4 × 0.5 mm^3^) were cut from the compression-molded plates. For measurements, a 4-point test fixture (gold contact wires with a distance of 16 mm between the source electrodes and 10 mm between the measuring electrodes) combined with a Keithley electrometer 6517A (Keithley Instruments, Cleveland, OH, USA) was used (filled symbols in the plots) in accordance with ASTM D 4496 [38]. For electrical resistivity values higher than 1 × 10^7^ Ohm cm, a Keithley 8009 Resistivity Test Fixture (Keithley Instruments, Cleveland, OH, US) based on ring electrodes was used for measurement on pressed circular plates (open symbols), according to ASTM D 257 [39]. In the plots, the geometric mean values and the standard deviations of 4–8 measurements are shown.

Uniaxial tensile tests were performed on injection-molded dogbone type (1A) specimens according to EN ISO 527-2/1A/50 [40] using a Universalprüfmaschine (Zwick GmbH & Co. KG, Ulm, Germany), which was equipped with a Multisens extensometer and a load cell of 20 kN. The tests were carried out at a crosshead speed of 50 mm/min. The values are the mean values of 8–10 samples. The E-modulus was measured at a crosshead speed of 1 mm/min.

The Charpy notched impact strength (notched a_cu_) was measured according to ISO 179/1eA [41] using a 4 J pendulum and a test velocity of 2.9 m/s, whereas the Charpy impact strength (a_cu_) was measured according to ISO 179/1eU, using a 15 J pendulum and a test velocity of 3.8 m/s. Whereas pure PC showed a hinge break, all composites had a complete sample break. All mechanical tests were performed after conditioning the samples at 23 °C @ 60% r. H. for at least 2 days.

## 3. Results

### 3.1. Influence of Throughput and Rotation Speed

#### 3.1.1. CNT Dispersion and Length

In the first set, during the preparation of PC composites filled with 3 wt.% MWCNTs, the rotation speed, throughput and feeding position were varied. The composites were investigated with regard to the MWCNT macrodispersion, as well as the electrical and mechanical properties, and for selected composites, the nanotube length distributions were also determined.

**Figure 2:**
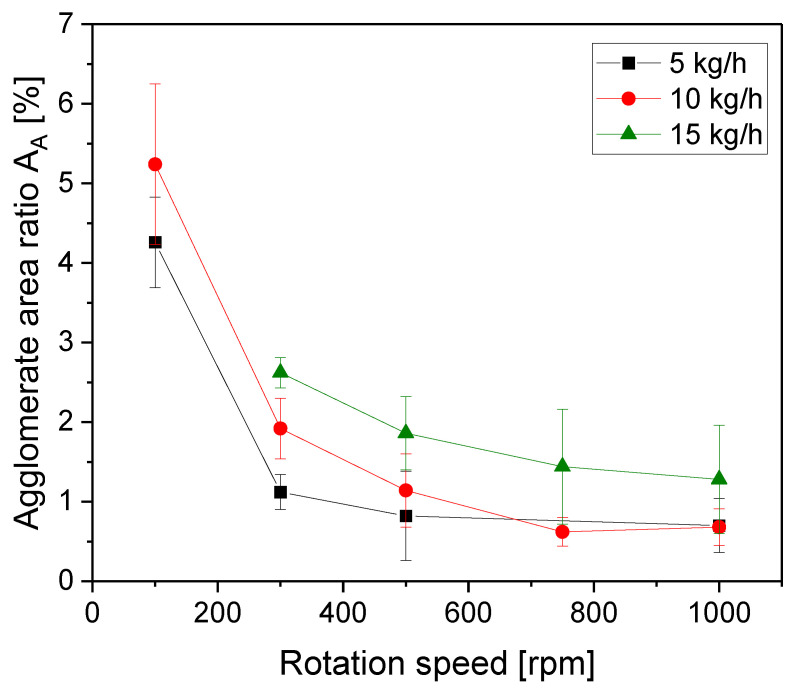
Agglomerate area ratio A_A_ vs. rotation speed for PC/3 wt.% MWCNT composites at different throughputs, with the feeding of Baytubes^®^ C150 P at the hopper, for screw No. SC-5.

**Figure 3:**
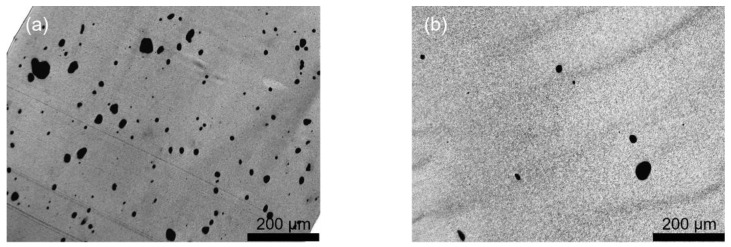
Light microscopy images of extruded PC/3 wt.% composites prepared at two processing conditions using screw SC-5, with the feeding of Baytubes^®^ C150 P at the hopper: (**a**) 100 rpm, 5 kg/h, SME = 0.16 kWh/kg, A_A_ = 4.3%; (**b**) 1000 rpm, 5 kg/h, SME = 0.90 kWh/kg, A_A_ = 0.7%.

The dependencies of the MWCNT agglomerate area ratio on the screw rotation speed at different throughputs are illustrated in Figure 2. Thereby, no composite could be produced at 15 kg/h and 100 rpm and no composite was manufactured at 5 kg/h and 750 rpm. Feeding of the MWCNTs was performed together with the PC pellets in the hopper. The area ratio was generally lower at a lower throughput, which was combined with a longer residence time and higher SME values. The largest decrease was between 50 and 250 rpm. At 5 kg/h throughput, the agglomerate area ratio leveled off starting at 500 rpm, at 10 kg/h, at 750 rpm, and at 15 kg/h, the increase in rotation speed resulted up to 1000 rpm in slight decreases. In addition, the light microscopy images of thin sections shown in Figure 3 illustrate the very different states of CNT macrodispersion at different rotation speeds. Significantly more remaining undispersed CNT agglomerates were seen when extrusion was performed at 100 rpm as compared to 1000 rpm. However, even at 1000 rpm, not all agglomerates were destroyed and some, even of a relatively large size, were still seen. 

**Figure 4:**
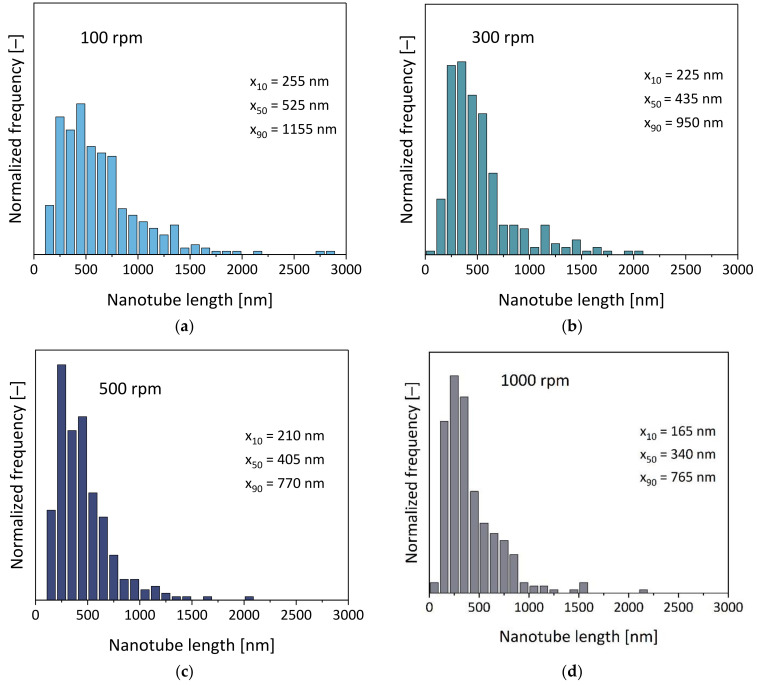
CNT nanotube length distributions at different rotation speeds for PC/3 wt.% Baytubes^®^ C150 P composites at 5 kg/h throughput, with the feeding of MWCNTs at the hopper, for screw No. SC-5: (**a**) 100 rpm, SME = 0.16 kWh/kg; (**b**) 300 rpm, SME = 0.39 kWh/kg; (**c**) 500 rpm, SME = 0.55 kWh/kg; (**d**) 1000 rpm, SME = 0.90 kWh/kg.

Furthermore, the nanotube length distributions in the composites were determined using TEM image analysis of the extracted CNTs and are shown for the example of different rotation speeds at a throughput of 5 kg/h in Figure 4. Compared to the initial length distribution of the pure MWCNT Baytubes^®^ (as shown in [1]) with x_10_, x_50_ and x_90_ values of 280 nm, 770 nm and 2410 nm, respectively, the length was significantly reduced after processing. Especially long tubes were shortened and the distributions shifted to lower length values with a higher proportion of CNTs with shorter lengths.

**Figure 5:**
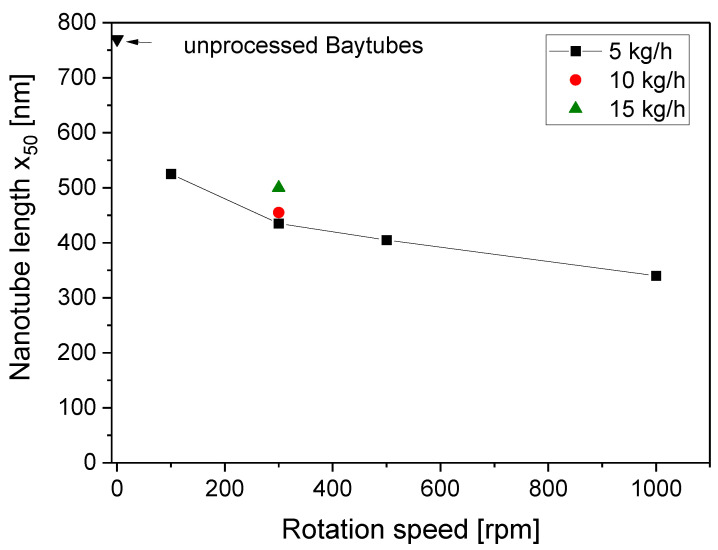
CNT nanotube length x_50_ vs. rotation speed for PC/3 wt.% MWCNT composites at different throughputs, with the feeding of Baytubes^®^ C150 P at the hopper, for screw No. SC-5.

The results in Figure 5 show the dependence of the x_50_ length values on rotation speed. As expected and described before by different authors [1,3,10,11,12,25,26,42], CNT shortening during the melt compounding took place. From comparing the different rotation speeds (at a throughput of 5 kg/h), a decrease in the nanotube lengths with the rotation speeds could be found. The mean x_50_ and x_90_ values decreased from 525 nm and 1155 nm (100 rpm) to 340 nm and 765 nm (1000 rpm), respectively. Therefore, a significant reduction in the aspect ratio was observed. At a constant rotation speed of 300 rpm, CNT shortening was more pronounced at a lower throughput, which correlated with the increasing SME values.

#### 3.1.2. Electrical Resistivity

**Figure 6:**
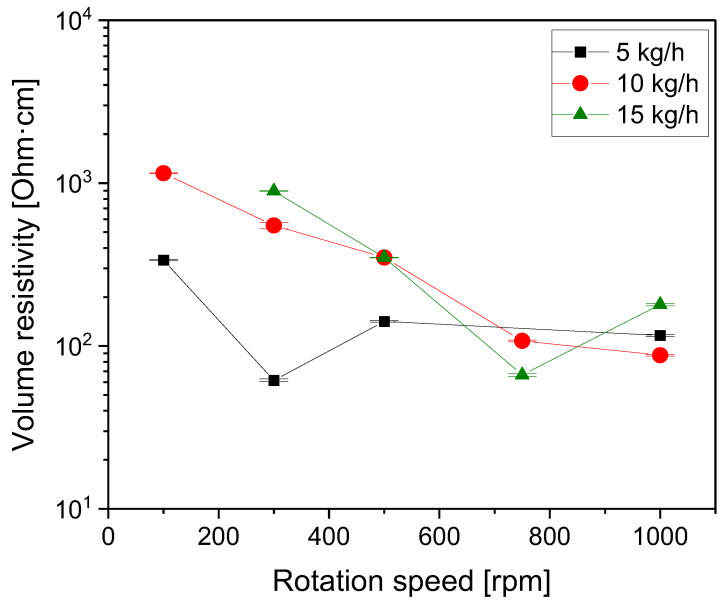
Electrical volume resistivity vs. rotation speed for PC/3 wt.% MWCNT composites at different throughputs, with the feeding of Baytubes^®^ C150 P at the hopper, for screw No. SC-5 (compression-molded plates).

The influence of rotation speed during compounding on the electrical properties was studied for composites prepared at different throughputs using screw No. SC-5. The volume resistivity values of the PC/3 wt.% composites showed a tendency to decrease with rotation speed (Figure 6). At 15 kg/h, a slight increase was seen when enhancing the rotation speed from 750 rpm to 1000 rpm, and at 5 kg/h, the value at 300 rpm showed the lowest resistivity value. Under this extrusion condition, the nanotube length x_50_ of 435 nm was measured, corresponding to a mean aspect ratio of 41 (compared to that of 73 for unprocessed nanotubes). The agglomerate area ratio A_A_ for this sample was 1.1%, which indicates that a worse dispersion compared to the sample produced at 1000 rpm with a ratio of 0.7% was achieved.

**Figure 7:**
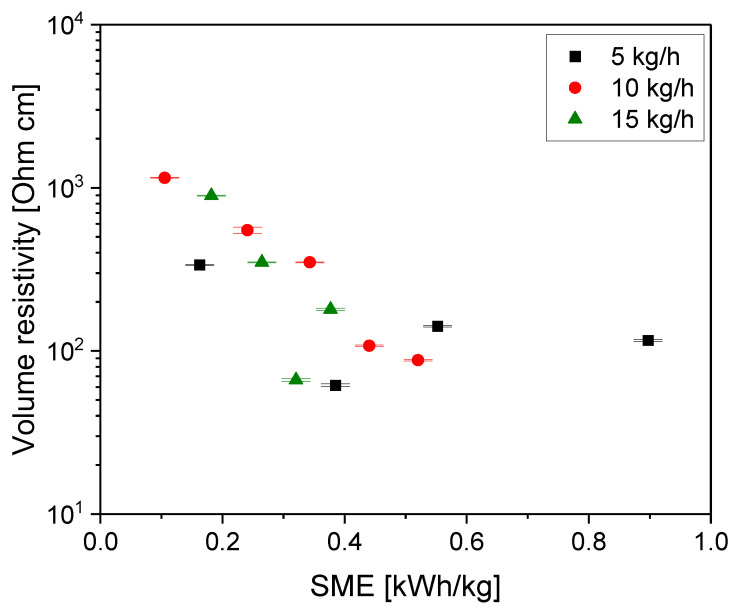
Electrical volume resistivity vs. SME for PC/3 wt.% MWCNT composites at different throughputs, with the feeding of Baytubes^®^ C150 P at the hopper, for screw No. SC-5 (compression-molded plates).

From plotting the values vs. the SME (Figure 7), the same general decreasing tendency was seen up to an SME of ca. 0.3 kWh/kg. The lowest resistivities were measured for composites produced with SMEs in the range of 0.3–0.4 kWh/kg; at a higher SME, a slight increase in electrical resistivity was observed. Interestingly, this SME value range was quite similar to the observed minimum in resistivity of PCL–MWCNT composites, which were produced at a small scale by variation in the rotation speed. There, the minimum was found at 0.47 kWh/kg [12]. Also in the PA6/MWCNT composites, which were also processed at a small scale by varying the rotation speed and mixing time, a minimum in resistivity was observed at an SME of 0.5 kWh/kg [1]. These findings reflect the balance between better CNT dispersion with increasing SME, resulting in reduced resistivity and the parallel ongoing reduction in the CNT length negatively affecting the resistivity. As both parameters are important for the formation of the conductive CNT network in the composites, minima of the resistivity were found.

**Figure 8:**
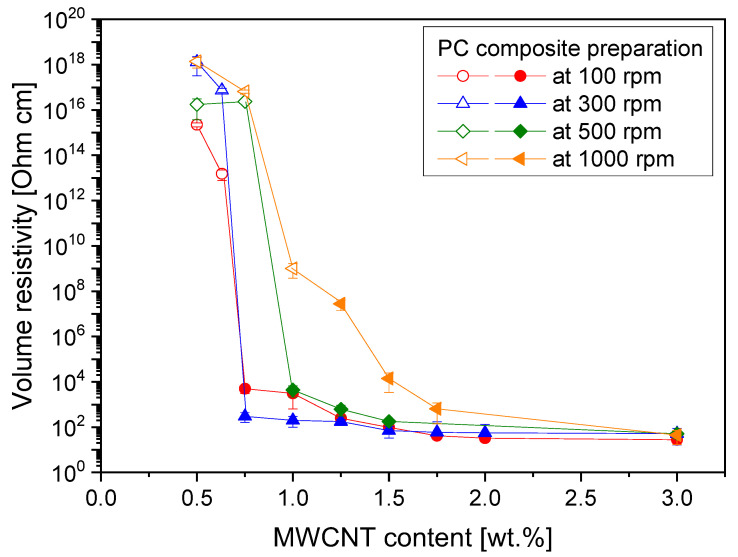
Electrical volume resistivity vs. CNT content for composites diluted using a microcompounder from extruded PC/3 wt.% Baytubes^®^ C150 P MWCNT composites prepared at different rotation speeds, with a throughput of 5 kg/h and using screw No. SC-5. Open symbols indicate measurements using Keithley 8009 and closed symbols with Keithley 6517A test configurations.

Since the electrical resistivity values for PC/3 wt.% MWCNT composites prepared at a throughput of 5 kg/h did not show a clear tendency, these composites were diluted to lower CNT contents using a microcompounder, and electrical percolation curves were determined, as shown in Figure 8. The lowest electrical percolation thresholds of 0.63 and 0.75 wt.% were determined for the composites prepared in LSE at 100 and 300 rpm, respectively. If the PC-3 wt.% composite was melt mixed in LSE at 500 rpm, the electrical percolation of the dilutions occurred between 1 and 1.25 wt.%. For the composites fabricated at the highest rotation speed of 1000 rpm, the highest electrical percolation threshold at 1 wt.% was found. For 300 rpm, the resistivity values at loadings up to 1.5 wt.% CNTs were the lowest, illustrating that this composite had the best balance between dispersion and CNT length to reach low electrical resistivities at low loadings. Thus, the lowest resistivity value observed at 3 wt.% loading at this rotation speed (see Figure 6 and Figure 7) was also reflected in the percolation behavior of the dilutions.

Additional melt rheological studies at 280 °C on the PC/3 wt.% MWCNT composites are shown in the Supplementary Materials in Figures S1–S5.

#### 3.1.3. Mechanical Properties

**Figure 9:**
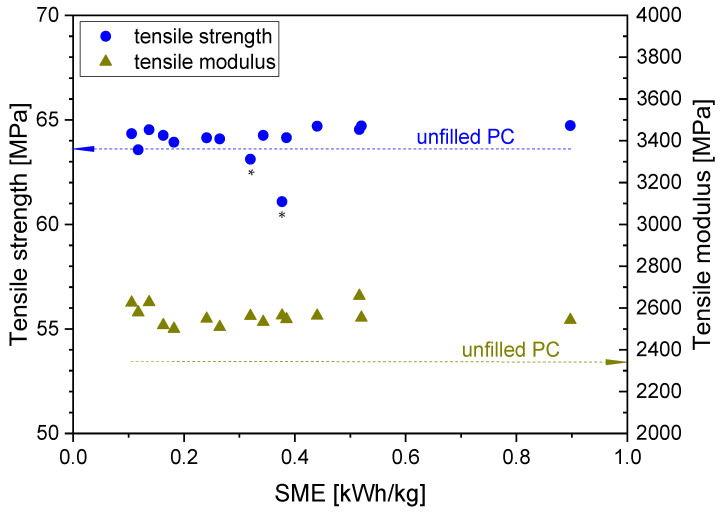
Tensile strength and tensile modulus vs. SME for PC/3 wt.% MWCNT composites at different rotation speeds and throughputs, with feeding at the hopper, for screw No. SC-5. The asterisk * marks the two samples with break partially or completely before reaching the yield point.

On the samples of this series, tensile tests were performed, and from the corresponding stress–strain curves, the elastic modulus, tensile strength and elongation at break were extracted (see Figure 9). Pure extruded PC showed a distinct yield point above which a cold drawing with nearly constant stress vs. strain was developed, which changed at about 90% strain into strain-hardening behavior. Most samples filled with 3 wt.% CNTs showed only cold-drawing behavior. Thus, the tensile strength as the maximum stress in the stress–strain curve in most samples represents the stress values at the yield point, whereas for pure PC, it is the stress at break. The samples prepared at 15 kg/h with 750 and 1000 rpm showed a break partially or completely before reaching the yield point and are therefore marked with an asterix *. When plotting the tensile strength of the composites vs. the SME applied during compounding, except for these two samples, slightly higher values than those measured for pure PC (63.7 ± 2.3 MPa) were found. Thereby the standard deviations of the tensile strength were between 0.1 and 3.6 MPa, those of the elastic modulus between 13 and 36 MPa. There was no significant dependence on the SME, a finding which was also reported by Mack et al. [21] for PC composites with 3 wt.% NC7000. The modulus was enhanced, on average by about 20%, as compared to pure extruded PC (2300 ± 12.4 MPa), again showing no significant differences when increasing the SME, similar to the report by Mack et al. [21].

**Figure 10:**
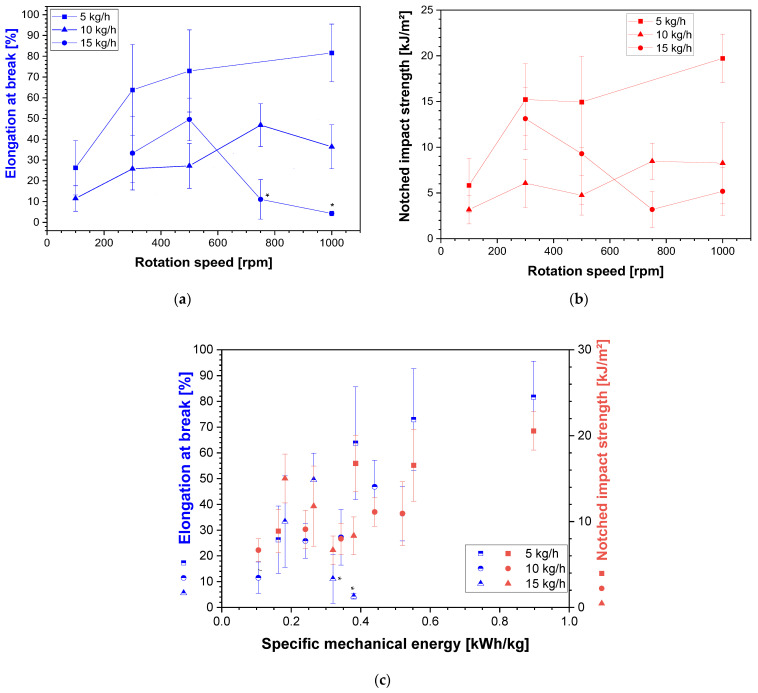
(**a**) Elongation at break and (**b**) notched impact strength vs. rotation speed for PC/3 wt.% MWCNT composites at different throughputs, and (**c**) elongation at break and notched impact strength vs. SME; with feeding at the hopper, for screw No. SC-5. The asterisk * marks the two samples with break partially or completely before reaching the yield point.

Figure 10a shows the elongation at break as obtained from the tensile tests and Figure 10b, the notched impact strength at different rotation speeds and different throughputs. For processed unfilled PC, the values of the elongation at break at 119 ± 12% and the notched impact strength at 80 ± 7 kJ/m^2^ were measured. With the incorporation of CNTs, both values decreased significantly. Both properties were strongly dependent on the state of CNT dispersion and network formation. Typically, a strong decrease in elongation at break is found in composites above the concentration of (electrical) percolation, as illustrated in [43] for PC/MWCNTs. The sharp decrease in elongation at break starting in the electrical percolation range seems to be connected to the restricted typical deformation behavior of the polymer chains as soon as a CNT network is interacting. At the CNT content of 3 wt.% selected in this study, all composites were conductive; however, they had different conductivities (as seen in Figure 6), illustrating different network structures.

When the rotation speed was increased, different developments were seen at the three studied throughputs. At the lowest throughput of 5 kg/h, an increase in elongation at break was found, even if the values had a relatively large standard deviation. At 10 kg/h, all values were lower than at 5 kg/h; however, up to 750 rpm, an increase with the rotation speed was noted. At 15 kg/h, an increase in elongation at break between 300 and 500 rpm was found at the level between the two other throughputs, followed by a strong decrease. For the values at higher rotation speeds, it has to be noted that the samples showed a break before reaching the yield point, such that the resulting type of stress–strain curve was different. This behavior may be attributed to a mechanical degradation of the PC by molecular weight reduction, which was not studied.

For the notched impact strength (Figure 10b), an increase with the rotation speed was again observed at 5 kg/h and 10 kg/h, whereas at 15 kg/h, a decreasing tendency was found, possibly again connected to PC degradation. Mack et al. [21] found a similar drastic decrease in the notched impact strength compared to the initial PC, and also an increase with the rotation speed, but no significant change with the throughput.

From plotting both properties vs. the SME, no clear picture was seen (Figure 10c). However, there was a generally increasing tendency for both values with the SME when disregarding the two values of elongation at break for the samples with changed stress–strain behavior (marked by asterix *).

### 3.2. Variation in Feeding Position

Furthermore, the influence of the feeding position was investigated at a rotation speed of 500 rpm, throughput of 5 kg/h and for screw No. SC-5. The feeding of CNT powder and PC granules into the hopper occurred as two separate streams. As CNTs, Baytubes^®^ C150 P and Nanocyl^TM^ NC7000 with a CNT content of 3 wt.% in a PC composite were used. The side feeder was located at an L/D of 14. The SME was nearly the same for all four preparation processes and was calculated to be around 0.5 kWh/kg. The results are summarized in Table 2.

It was found that feeding in the hopper for Baytubes^®^ C150 P resulted in better CNT dispersion, as indicated by a lower value of the agglomerate area ratio A_A_. In contrast, with MWCNT NC7000, feeding into the side feeder led to significantly better CNT dispersion. Interestingly, Müller et al. already described the same tendencies when incorporating both kinds of MWCNTs into PP [17]. The differences can be explained by the different agglomerate structures in both MWCNT materials. Compared with side feeding, the incorporation of CNTs at the hopper at the beginning of the extrusion process led to a significantly longer residence time of the CNTs in the polymer melt and induced, especially in the melting zone of the extruder, higher shear stresses. The more compact Baytubes^®^ C150 P material required higher shear stresses for the dispersion of its agglomerates, whereas MWCNT NC7000 has a less dense and combed yarn structure [18] and needed a comparatively lower energy input for dispersion. Thus, feeding into the already molten polymer is favorable in the context of dispersion.

**Figure 11:**
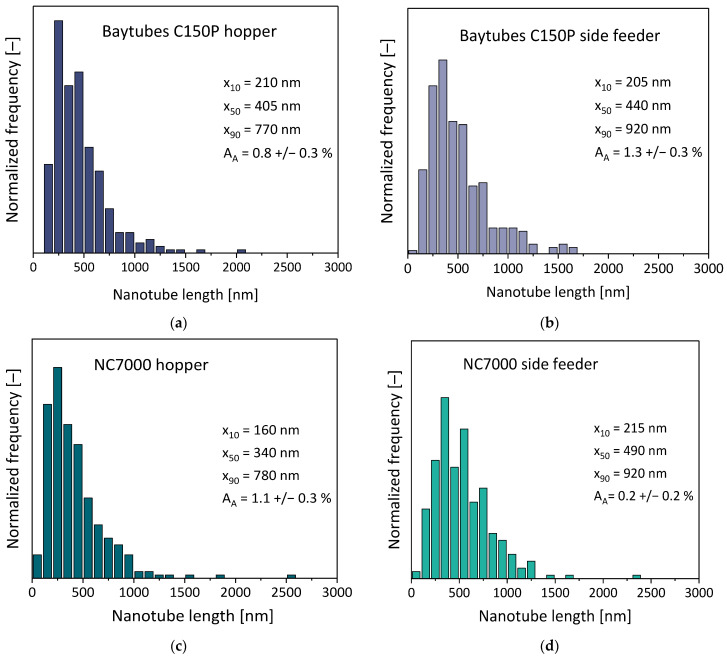
MWCNT length distributions for PC/3 wt.% MWCNT composites prepared using different feeding positions, at 500 rpm and 5 kg/h, for screw No. SC-5: (**a**) Baytubes^®^ C150 P at the hopper; (**b**) Baytubes^®^ C150 at the side feeder; (**c**) Nanocyl^™^ NC7000 at the hopper; (**d**) Nanocyl^™^ NC7000 at the side feeder.

For both kinds of MWCNTs, the value of the electrical volume resistivity ρ was lower for the composites with poorer CNT dispersion, which can be interpreted in the context of the shortening of the CNT length during melt processing. The CNT shortening was more pronounced by the feeding of CNTs in the hopper as also clearly seen in the CNT length distribution of the extracted CNTs in Figure 11. CNT incorporation using the side feeder led both MWCNT types to have slightly higher values of tensile strength (σ_max_), and significantly higher values of elongation at break ε_Break_ and impact as well as notched impact strength a_CU_ of their composites. The values of the tensile modulus E_t_ and strength σ_max_ were not influenced by the feeding position. It can be summarized that the side feeding led to slightly better electrical and mechanical properties for both kinds of MWCNTs.

### 3.3. Influence of Screw Configuration

**Figure 12:**
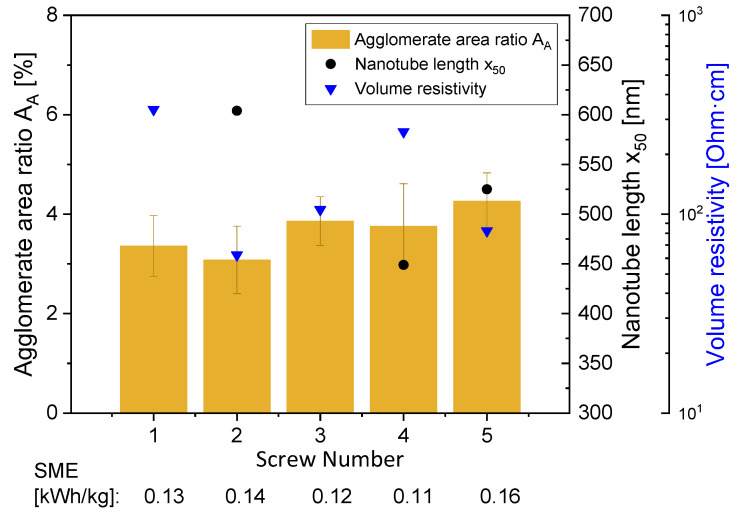
Electrical volume resistivity, agglomerate area ratio and CNT length x_50_ at different screw configurations for PC/3 wt.% Baytubes^®^ C150 P composites (hopper feeding, 100 rpm, 5 kg/h). Standard deviations for volume resistivity values are given in Table 3.

In the second set of experiments, the selected screw configuration (under otherwise constant set extrusion parameters) had only a very low impact on the different studied properties of PC/3 wt.% Baytubes^®^ C150 P composites, as illustrated in Table 3. This was also due to the fact that the SME did not differ much (0.11 to 0.16 kWh/kg, see Figure 12) under the selected conditions of a rotation speed of 100 rpm and throughput of 5 kg/h. The agglomerate area ratio A_A_ varied between 3.1 and 3.9%. The mechanical properties did not differ much, but had relatively large standard deviations. Similar results of a low influence of the screw configuration on different composite properties were also found in a study on PLA/MWCNT NC7000 composites by Villmow et al. [15].

**Figure 13:**
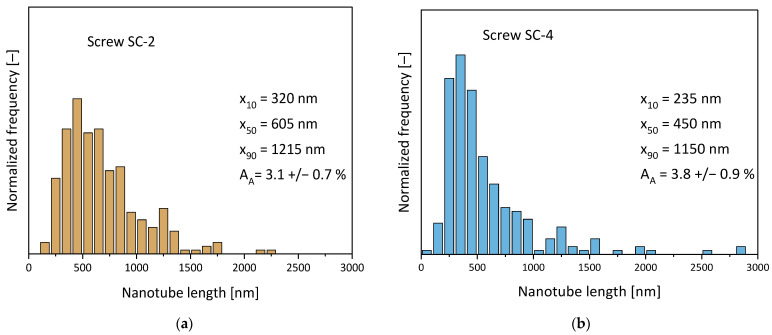
CNT length distributions for PC/3 wt.% Baytubes^®^ C150 P composites prepared using different screw configurations, at 100 rpm and 5 kg/h, with feeding at the hopper: (**a**) screw SC-2, SME = 0.14 kWh/kg; (**b**) screw SC-4, SME = 0.11 kWh/kg (for SC-5, see Figure 4a).

CNT shortening was slightly less pronounced when using the dispersive screw SC-2 compared to the distributive screw and that with the longer screw length. A comparison of the CNT length distribution of MWCNTs extracted from the composites prepared using SC-2 and SC-4 is given in Figure 13, and that for SC-5 is represented by Figure 4a. The MWCNTs extracted after applying SC-4 showed the highest population of nanotubes in the lower length classes, even if some CNTs with higher lengths were also measured. The distributions were more shifted to higher CNT length classes for SC-5 and even more for SC-2.

### 3.4. Relation between SME, Dispersion and Nanotube Length

**Figure 14:**
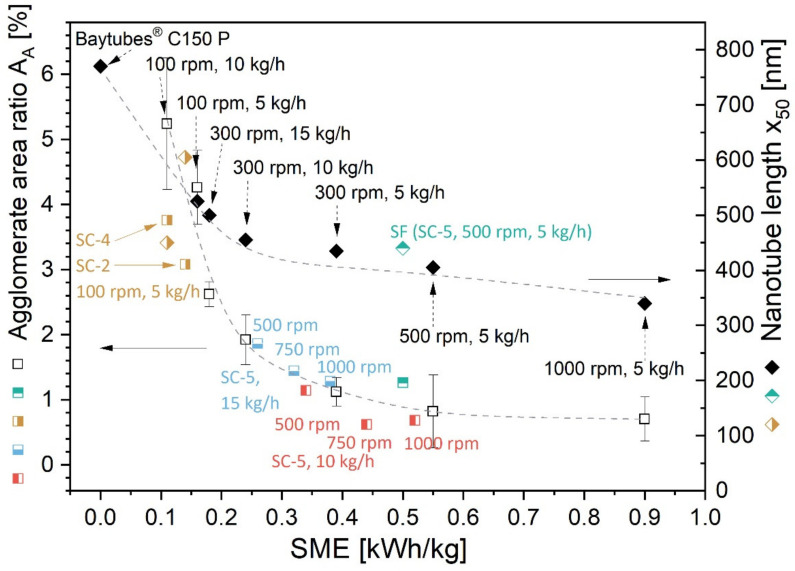
Agglomerate area ratio A_A_ and nanotube length x_50_ vs. SME for PC/3 wt.% MWCNT composites at different rotation speeds, throughputs, MWCNT feedings and screw designs. Values (in black) for screw SC-5 with directly feeding Baytubes^®^ C150 P replotted with permission from Ref. [1] RSC 2013. Newly added values (direct feeding) in different colors (standard deviations of A_A_ shown before): brown symbols screws SC-2 and SC-4 at 100 rpm, 5 kg/h; light blue symbols, screw SC-5 at 500–1000 rpm, 15 kg/h; red symbols, screw SC-5 at 500–1000 rpm, 10 kg/h; and green symbols, side feeding (SF) screw SC-5 at 500 rpm, 5 kg/h. The lines are meant to guide the eyes.

In order to summarize all the results using the variation in rotation speed, throughput, feeding position and screw configuration concerning their influence on dispersion and MWCNT shortening, Figure 14 shows the dependence of the agglomerate area ratio A_A_ and the CNT length x_50_ on the applied SME for all the composites with 3 wt.% MWCNTs Baytubes^®^ C150 P. Interestingly, the data fit together in a general tendency. The plot also contains the mixing conditions. Looking at the effect of processing conditions on the CNT macrodispersion, it was found that the agglomerate area ratio A_A_ decreased with increasing SME and leveled off at an SME input of around 0.4 kWh/kg. A reduction in A_A_ from 5.2% at an SME of 0.11 kWh/kg to an A_A_ of 0.7% at 0.90 kWh/kg was found. The length reduction was also significant, and at the highest used SME of 0.90 kWh/kg, the length was only about 44% of the original length value of the pure Baytubes^®^ material (corresponding to a mean aspect ratio of 32), whereas at an SME of 0.16 kWh/kg, it was about 64% (aspect ratio of 47). There was a significant length decrease up to about 0.24 kWh/kg and a much lower decrease when further enhancing the SME. In summary, increasing the SME above about 0.4 kWh/kg did not improve dispersion significantly and the final length of the CNTs of about 350 nm seemed to be reached.

### 3.5. CNT Shortening at Different Loadings

**Figure 15:**
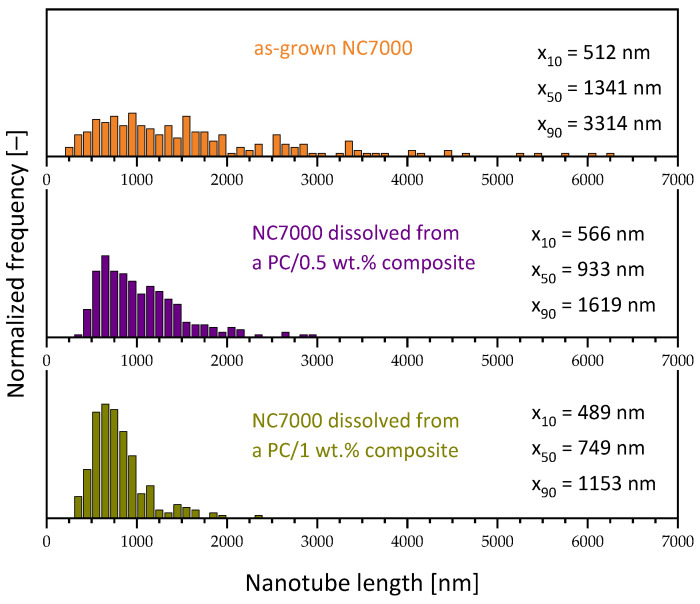
CNT length distributions of as-grown Nanocyl^TM^ NC7000 MCWNTs (adapted from [11] ELSEVIER 2011) and PC (Makrolon^®^2205) composites filled with 0.5 and 1 wt.% NC7000 prepared at Xplore 15 (260 °C, 250 rpm, 15 min, PM2) (adapted from [26] JOHN WILEY AND SONS 2017).

Whereas in the small-scale mixing of PC (grade Makrolon^®^ 2205, low melt viscosity) with Nanocyl^TM^ NC7000 MWCNTs, results comparing 0.5 and 1.0 wt.% were already reported [26] (Figure 15), no results could be found for LSE. As expected, under constant mixing conditions (here shown for the parameter set 2, with mixing at 5 min, at 250 rpm, using a 15 cm^3^ DSM Xplore microcompounder), the length shortening was significantly higher at the higher loading. At 1.0 wt.% CNTs, the x_50_ value decreased from 1341 nm of the initial MWCNTs to 749 nm, which equals a reduction to 56% of the initial length. At 0.5 wt.% of MWCNT loading, the reduction was only to 70%.

**Figure 16:**
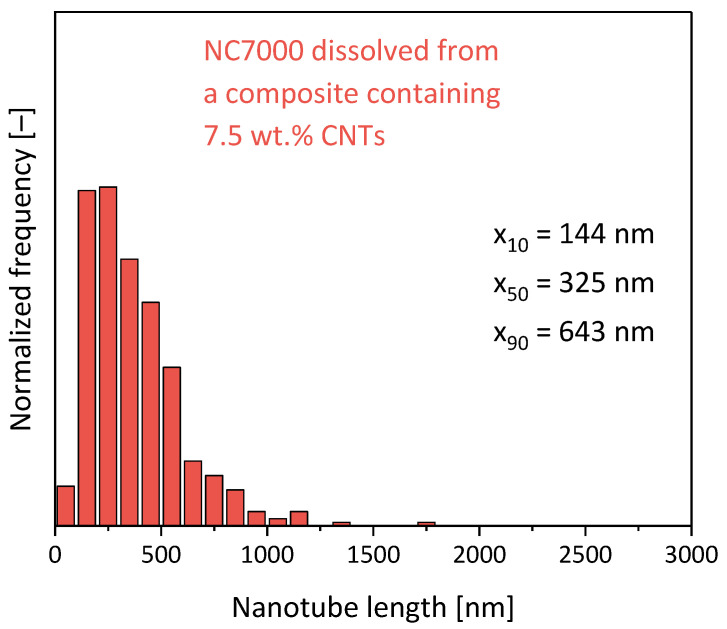
CNT length distributions of NC7000 MWCNTs dissolved from a PC/7.5 wt.% NC7000 masterbatch (prepared using Berstorff ZE25, SC-5, 260 °C, 5 kg/h, 500 rpm).

**Figure 17:**
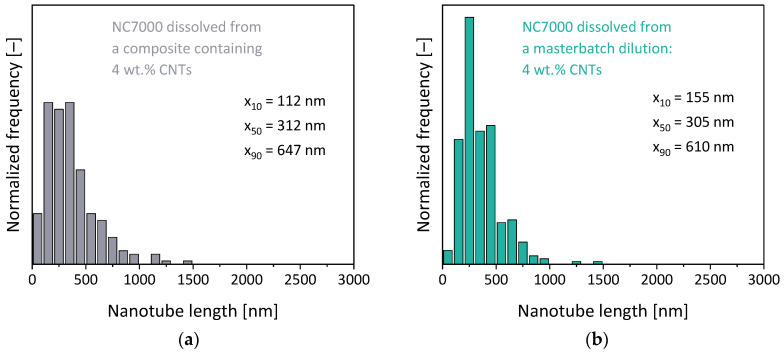

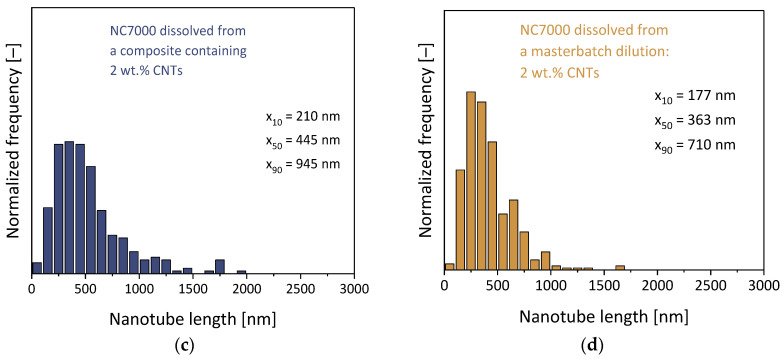
CNT length distributions of (**a**) directly compounded PC/4 wt.% NC7000; (**b**) a composite prepared by dilution of a masterbatch (7.5 wt.%) to 4 wt.%; (**c**) directly compounded PC/2 wt.% NC7000; (**d**) a composite prepared by masterbatch dilution to 2 wt.% (Berstorff ZE25, SC-5, 260 °C, 5 kg/h, 500 rpm).

To see that effect in LSE, in which higher shear stresses and more pronounced shortening may have been expected, composites with the same type of MWCNTs in another type of PC were prepared in a third set. A masterbatch with 7.5 wt.% Nanocyl^TM^ NC7000 MWCNTs was diluted to 2 wt.% and 4 wt.% composites, and the CNT lengths of those composites were compared to those of composites prepared by direct incorporation of the MWCNTs under the same conditions, as used for masterbatch preparation and dilution. From comparing the masterbatch and the two composites with lower loadings, all prepared under the same set conditions by direct MWCNT incorporation (Figure 16 and Figure 17), it was seen that the CNT length decreased to about the same value when using 7.5 wt.% or 4.0 wt.% (ca. 24%, mean aspect ratio of 32). By incorporating 2 wt.% directly, the shortening was lower and resulting in x_50_ values of 33% of the initial length (aspect ratio of 44). It was also seen that the shortening was much higher in the LSE compared to the small-scale mixing studied before and discussed above. The composites prepared by dilution of the masterbatch, which had experienced shear stresses of extrusion twice, showed even higher shortening, especially seen at 2 wt.% loading, where the x_50_ value was only 27% (aspect ratio of 36) of the value of the initial MWCNTs.

This illustrates a significant and much higher relative shortening compared to the MWCNTs of the Baytubes^®^ type which were studied before and had a lower initial length. Interestingly the CNT length shortening seems to have leveled off to length values of about 300 to 400 nm (related to x_50_), a value which was also found for other CNT materials, matrix polymers and mixing conditions [1,10,12,25]. It may be concluded that longer CNTs show more pronounced shortening than shorter ones, a finding which was already reported by Rhue et al. [44] for ball-milled CNTs and by Krause et al. [25] for differently ball-milled CNTs incorporated in PC.

The comparison between the lengths at different loadings also showed that there seemed to be a problem with the selected dissolving method in extracting the CNTs properly especially at higher MWCNT loadings, where the CNTs were much more agglomerated. Such agglomerates may contain long nanotubes to a particular extent. As such agglomerates are not dispersed during the mild ultrasound treatment they could have been neglected due to their sedimentation in the dispersion by the applied preparation method for the TEM grids. Thus, some of the longer nanotubes may not have been available for the quantitative TEM analysis. This is indicated by the x_50_ and the x_90_ length values measured in the diluted composite with 2 wt.%, where a higher length was determined than in the starting masterbatch with 7.5 wt.% MWCNTs.

## 4. Summary and Conclusions

For composites of PC with MWCNTs, the presented results show that there is a strong influence of the twin screw extrusion conditions at the laboratory scale during their preparation on the achieved state of filler dispersion and the nanotube length. Both properties influence the electrical resistivity of the composites and the mechanical properties. The specific mechanical energy (SME) introduced during compounding is a good measure to quantify the effect of variations in screw speed, throughput, feeding position and screw profile on the mentioned composite properties.

On composites with 3 wt.% MWCNTs of the Baytubes^®^ C150 P type, all being in the electrically conductive range above the electrical percolation threshold, it was shown that dispersion was enhanced with rotation speed and throughput, feeding the material in the hopper, and using a dispersive screw configuration. As measured using transmission light microscopy, the agglomerate area ratio varied between 5.2 and 0.6%, indicating that some undispersed MWCNT material was still existent in the extrudates. The dispersion increased with the SME being up to about 0.4 kwh/kg and then leveled off. The initial MWCNT length of 770 nm decreased with the rotation speed, up to values of 340 nm, whereby the decrease was more pronounced at lower throughputs. This decrease was stronger when the MWCNT material was fed to the hopper together with the PC granules. The latter result was also found for MWCNTs of the Nanocyl™ NC7000 type, whereby the absolute length decrease was much higher (up 25% of the initial length) for Nanocyl™ NC7000 as compared to that of the Baytubes^®^ C150 P material (up to 53%). Thereby, the final lengths for both MWCNT types were in the range of 340 to 500 nm (based on the x_50_ values of the length distributions obtained from the TEM images of the dissolved MWCNTs), which seems to be a value at which the shortening leveled off. The electrical volume resistivity of compression-molded samples tended to decrease with rotation speed and the SME with a slight minimum in the range of 0.3 to 0.5 kWh/kg. This variation was between 60 and 1200 Ohm∙cm, meaning more than one decade. It was nearly independent of screw configuration. The named SME range seems to be an optimum between good dispersion and not too harsh CNT shortening. Concerning the mechanical properties, the tensile modulus and strength were not significantly influenced by the processing conditions, whereas the elongation at break and notched impact strength tended to increase with the SME.

In addition, it was shown in the example of composites with MWCNTs of the Nanocyl™ NC7000 type that the MWCNT length reduction also depended on the concentration of the nanotubes in the mixing recipe. The shortening tended to be higher at a higher CNT loading and more pronounced if a masterbatch dilution process was used as compared to the direct CNT incorporation to obtain the same MWCNT concentration.

This study indicates that the effect of carbon nanotube shortening is significant in the twin screw extrusion of composites and needs to be considered when optimizing processing conditions to achieve materials with high electrical performance and/or high mechanical properties. The shown effects may be even more pronounced when composites with (lower) concentrations nearer to the electrical percolation threshold are considered, as the nanotube networks are much more sensitive to small structural differences when they are looser. These results can provide a basis for giving recommendations for the selection of proper processing conditions during composite production. However, the shaping conditions into final parts, e.g., by injection molding, also need to be considered. Here, it was interesting to study to what extent the different properties obtained in the granules after composite production could be modified by the injection molding conditions. In this context, attention should also be paid to the changes in the molecular weight of the matrix polymers involved in this processing.

## Figures and Tables

**Table 1:** Screw configurations (according to the screw designs used in Ref. [16]).

Screw No.	L/D Ratio	Screw Design
SC-1	36	Dispersive: 4 kneading blocks and 2 back-conveying elements
SC-2	36	Dispersive: 4 kneading blocks and 6 back-conveying elements
SC-3	36	Distributive: 3 mixing elements and 2 back-conveying elements
SC-4	36	Distributive: 3 mixing elements and 8 back-conveying elements
SC-5	48	Distributive: 5 mixing elements and 8 back-conveying elements

**Table 2:** Properties of composites (PC/3 wt.% MWCNT) produced under variation in the feeding position.

Feeding	A_A_ [%]	ρ [Ohm·cm]	CNT Length x_50_ [nm]	E_t_ [GPa]	σ_max_ [MPa]	ε_Break_ [%]	a_cu_ [kJ/m^2^]	Notched a_cu_ [kJ/m^2^]
Baytubes^®^ C150 P								
hopper	0.8 ± 0.3	42.4 ± 1.6	405	2.7	64.5 ± 0.2	26.1 ± 6.9	170.7 ± 90.7	6.9 ± 1.4
side feeder	1.3 ± 0.8	11.6 ± 1.1	440	2.7	64.9 ± 0.2	73.6 ± 14.3	329.2 ± 12.1	8.7 ± 1.9
Nanocyl^TM^ NC7000								
hopper	1.1 ± 0.3	17.6 ± 1.2	337	2.7	65.7 ± 1.4	11.2 ± 6.1	123.0 ± 84.9	7.3 ± 1.9
side feeder	0.2 ± 0.2	24.2 ± 1.4	494	2.7	66.7 ± 0.1	30.6 ± 11.6	240.2 ± 112.1	10.1 ± 2.9

**Table 3:** Properties of composites (PC/3 wt.% Baytubes^®^ C150 P MWCNTs) produced under variations in the screw configuration and feeding at the hopper, with a rotation speed of 100 rpm and throughput of 5 kg/h.

Screw	A_A_ [%]	ρ [Ohm·cm] ^#^	CNT Length x_50_ [nm]	E_t_ [GPa]	σ_max_ [MPa]	ε_Break_ [%]	a_cu_ [kJ/m^2^]	Notched a_cu_ [kJ/m^2^]
SC-1	3.4 ± 0.6	62.3 ± 1.2		2.6	51.4 ± 1.1	20.1 ± 12.6	312.3	9.1 ± 2.1
SC-2	3.1 ± 0.7	105.5 ± 1.6	604	2.6	50.9 ± 0.7	25.4 ± 9.6	293.9 ± 53.3	10.6 ± 3.3
SC-3	3.9 ± 0.5	259.8 ± 1.6		2.6	50.8 ± 2.2	38.8 ± 16.8	293.7 ± 48.1	8.1 ± 2.5
SC-4	3.8 ± 0.9	82.5 ± 1.5	450	2.6	50.9 ± 0.7	23.5 ± 7.6	289.2 ± 104.9	9.5 ± 3.1
SC-5	4.2 ± 0.5	297.2 ± 3.4	525	2.6	50.8 ± 0.8	31.6 ± 13.1	318.2 ± 2.4	10.1 ± 2.3

# on compression-molded plates.

## Data Availability

The TEM images of MWCNTs detached from PC composites to determine the MWCNT length distributions are openly available in Zenodo at https://doi.org/10.5281/zenodo.11400466, Ref. [37]. All other raw data are available in Zenodo at https://doi.org/10.5281/zenodo.13309199, Ref. [45]. This reference also contains the data sheets for the materials used.

## Supplementary Materials

The following supporting information can be downloaded at: https://www.mdpi.com/article/10.3390/polym16192694/s1, Figure S1: Complex melt viscosity |Eta*| vs. frequency for processed PC and PC/3 wt.% MWCNT composites prepared in LSE at different rotation speeds; Figure S2: Storage modulus G’ vs. frequency for PC/3 wt.% MWCNT composites prepared in LSE at different rotation speeds; Figure S3: Loss modulus G″ vs. frequency for PC/3 wt.% MWCNT composites prepared in LSE at different rotation speeds; Figure S4: Tan delta vs. frequency for PC/3 wt.% MWCNT composites prepared in LSE at different rotation speeds; Figure S5: Storage modulus G′ vs. loss modulus G″ for processed PC and PC/3 wt.% MWCNT composites prepared in LSE at different rotation speeds.
