# Supplementary material for: Influence of Twin Screw Extrusion Conditions on MWCNT Length and Dispersion and Resulting Electrical and Mechanical Properties of Polycarbonate Composites
Source: Polymers (Basel). 2024 Sep 24;16(19):2694. doi: 10.3390/polym16192694 (PMC11478397; doi:10.3390/polym16192694)
Supplement: Supplementary file 1 [file polymers-16-02694-s001.zip › polymers-3160303-supplementary.pdf]

Influence of Twin Screw Extrusion Conditions on MWCNT Length and Dispersion and Resulting Electrical and Mechanical Properties of Polycarbonate Composites

Petra Pötschke [1,*], Tobias Villmow [1], Beate Krause [1] and Bernd Kretzschmar [1]

[1]Leibniz-Institut für Polymerforschung Dresden e.V., Hohe Str. 6, 01069 Dresden, Germany; krause-beate@ipfdd.de (B.K.), bkretzsch@online.de (B.Kr.)
*Correspondence: poe@ipfdd.de; Tel.: +49 351 4658 395 (P.P.)

Supplementary Materials

Melt rheological studies were performed on extruded PC/ 3 wt.% MWCNT (Baytubes® C150P) composites prepared under laboratory scale Extrusion (LSE) at different rotation speeds, 5 kg/h throughput and using screw no. SC-5 (see main text, chapter 3.1.). The pure PC granules were processed under the same conditions using a screw speed of 500 rpm.

The measurements were done on compression molded disks with a diameter of 25 mm and a thickness of about 1 mm, prepared at 280°C for 1 min pressing time in a Weber hot press (Model PW 40 EH, Paul Otto Weber GmbH, Remshalden, Germany). An ARES oscillation rheometer (TA instruments, New Castle, DE, US) was applied under nitrogen atmosphere and a melt temperature of 280°C was used for the frequency sweeps. The sweeps were performed from 0.063 rad/s to 100 rad/s as upward and downward sweeps, and the downward sweeps were used for the graphs. A strain of 10 % was applied.

Rheological data of PC/ 3 wt.% MWCNT (Baytubes® C150 P) at 280°C

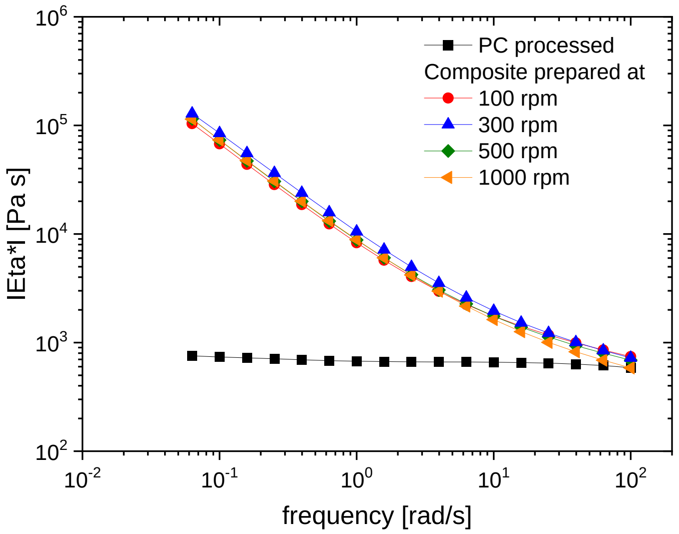

Fig. S1: Complex melt viscosity IEta*I vs. frequency for processed PC and PC/ 3 wt.% MWCNT composites prepared in LSE at different rotation speeds.

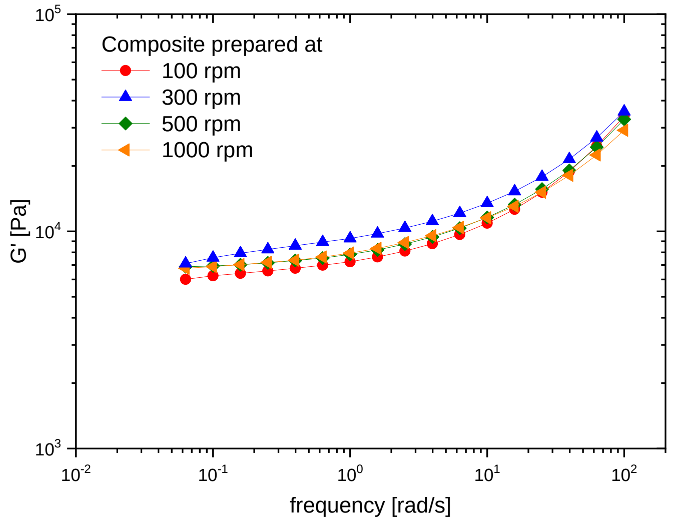

Fig. S2: Storage modulus G’ vs. frequency for PC/ 3 wt.% MWCNT composites prepared in LSE at different rotation speeds

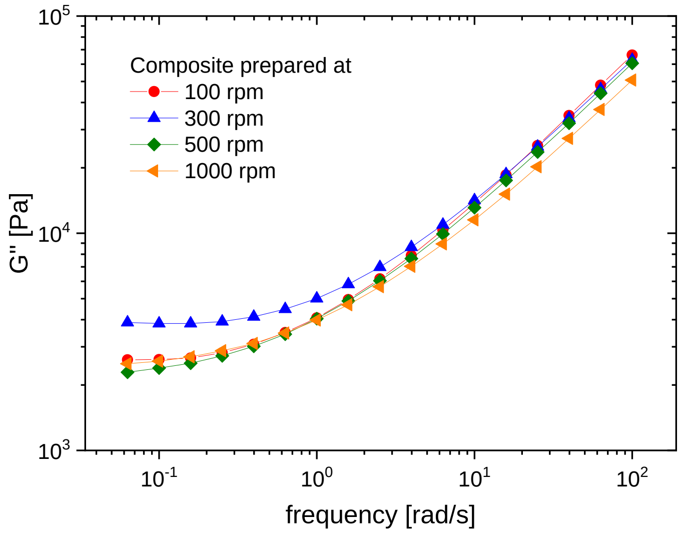

Fig. S3: Loss modulus G’’ vs. frequency for PC/ 3 wt.% MWCNT composites prepared in LSE at different rotation speeds

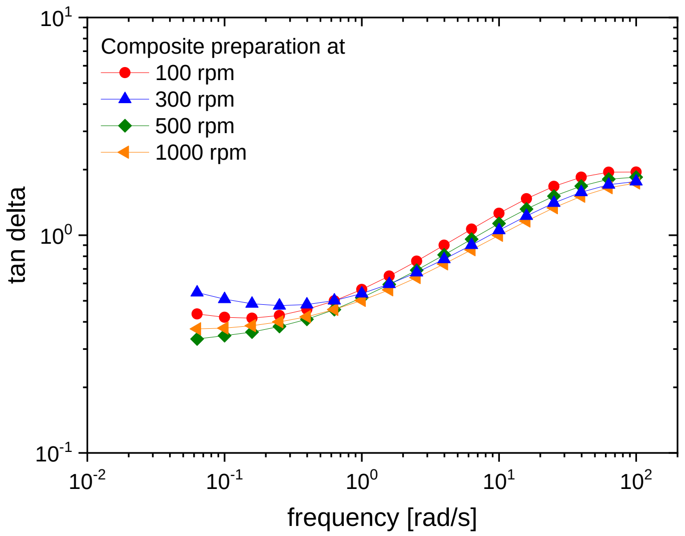

Fig. S4: Tan delta vs. frequency for PC/ 3 wt.% MWCNT composites prepared in LSE at different rotation speeds

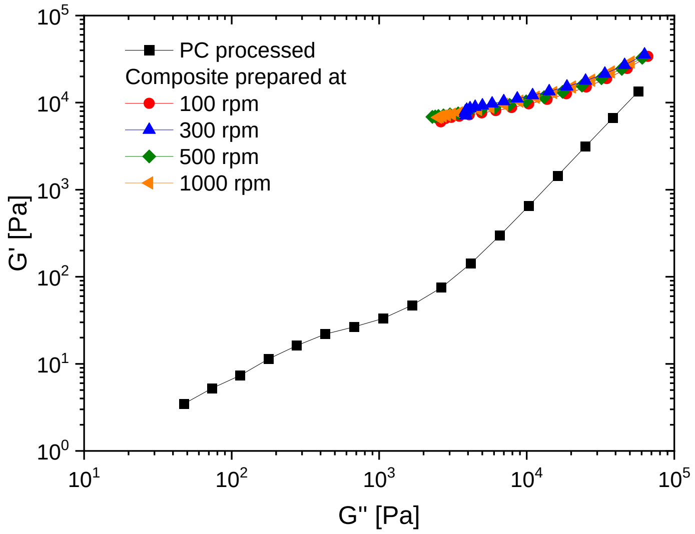

Fig. S5: Storage modulus G' vs. loss modulus G'' for processed PC and PC/ 3 wt.% MWCNT composites prepared in LSE at different rotation speeds

The differences between the rheological properties of the composites with 3 wt.% MWCNTs prepared at different rotation speeds are only marginal. The composite prepared at 300 rpm shows slightly higher storage modulus G' and loss modulus G'', resulting in higher complex viscosity IEta*I than those prepared at the lower or higher rotation speed. This may be attributed to a suitable combination of already good CNT dispersion and not too high length reduction with not too high molecular weight degradation of the polymer matrix. The maximum in these values corresponds with the lowest electrical resistivity value observed at the rotation speed of 300 rpm (see Figure 6), illustrating an electrical network structure which results in as well highest electrical as rheo-mechanical performance.

A significant influence is seen concerning the comparison of pure processed PC and the composites. All composites show a strong increase in the complex viscosity with decreasing frequency and much higher storage than loss modulus than processed PC indicating clearly a percolated CNT network structure.

Acknowledgments: We thank Miss Anna Ivanov (IPF Dresden) for performing the rheological measurements.
